# Anti-dengue activity of super critical extract and isolated oleanolic acid of *Leucas cephalotes* using in vitro and in silico approach

**DOI:** 10.1186/s12906-021-03402-2

**Published:** 2021-09-08

**Authors:** Sulochana Kaushik, Lalit Dar, Samander Kaushik, Jaya Parkash Yadav

**Affiliations:** 1 Department of Genetics, Maharshi Dayanand University, Rohtak, Haryana 124001 India; 2 Department of Microbiology, All India Institute of Medical Sciences, Delhi, 110029 India; 3 Centre for Biotechnology, Maharshi Dayanand University, Rohtak, Haryana 124001 India

**Keywords:** *Leucas cephalotes* SFE extract, Oleanolic acid, HPTLC, NMR, Anti-dengue, molecular docking

## Abstract

**Backgrounds:**

*Leucas cephalotes* is a common ethnomedicinal plant widely used by traditional healers for the treatment of Malaria and other types of fever. Oleanolic acid and its derivatives have been reported for various types of pharmacological activities, such as anti-inflammatory, antioxidant, anticancer, hepatoprotective, anti-HIV and anti-HCV activity.

**Methods:**

*L.cephalotes* plant extracts were prepared by supercritical fluid extraction (SFE) method and oleanolic acid was isolated by preparatory thin-layer chromatography. The compound was identified and characterize by using ultraviolet-visible spectroscopy (UV-VIS), Fourier transform infra-Red spectroscopy (FT-IR) and high-performance thin-layer chromatography (HPTLC). The structure of the compound was elucidated by proton nuclear magnetic resonance (^1^HNMR) and carbon nuclear magnetic resonance (^1^CNMR) and the purity checked by differential scanning calorimetry (DSC). The MTT assay was used to determine the toxicity of plant extract and oleanolic acid using a microplate reader at 595 nm. The anti-dengue activity of plant extract and oleanolic acid was tested in vitro and in silico using real-time RT-PCR.

**Results:**

The optimum yield of the extract was obtained at 40 °C temperature and 15Mpa pressure. The maximum non-toxic dose (MNTD) of plant extract and oleanolic acid were found as 46.87 μg/ml and 93.75 μg/ml, respectively in C6/36 cell lines. UV spectrophotometer curve of the isolated compound was overlapped with standard oleanolic acid at 232 nm. Superimposed FT-IR structure of the isolated compound was indicated the same spectra at 3433, 2939, 2871, 1690, 1500,1463, 1387, 1250, 1209, 1137 and 656 position as per marker compound. HPTLC analysis showed the retention factor of *L. cephalotes* extract was 0.19 + 0.06 as similar to the standard oleanolic acid chromatogram. The NMR structure of the isolated compound was identified as similar to the marker oleanolic acid structure. DSC analysis revealed the purity of isolated oleanolic acid was 98.27% with a melting point of 311.16 °C. Real-time RT PCR results revealed that *L. cephalotes* supercritical extract and isolated oleanolic acid showed 100 and 99.17% inhibition against the dengue − 2 virus when treated with MNTD value of plant extract (46.87 μg/ml) and the test compound (93.75 μg/ml), respectively. The molecular study demonstrated the binding energy of oleanolic acid with NS1and NS5 (non-structural protein) were − 9.42 & -8.32Kcal/mol, respectively.

**Conclusions:**

The SFE extract *L. cephalotes* and its active compound, oleanolic acid inhibiting the activity of dengue-2 serotype in the in vitro and in silico assays. Thus, the *L.cephalotes* plant could be an excellent source for drug design for the treatment of dengue infection.

**Supplementary Information:**

The online version contains supplementary material available at 10.1186/s12906-021-03402-2.

## Background

Dengue fever is a major health issue and there are no antiviral medicines available to treat it. The number of dengue cases is increased by more than 6 fold, from < 0.5 million in 2010 to over 3.34 million in 2016 [1]. According to the National Vector Borne Disease Control Programme (NVBDCP), 136,422 dengue cases were reported and 132 death from all over India in the year 2019 [2]. The dengue is caused by the dengue virus (family *Flaviviridae*, genus *Flavivirus*) with four serotypes, DENV1–4 [3, 4]. Dengue-2 is a more lethal serotype than the others. The first outbreak of dengue hemorrhagic fever (DHF) and dengue shock syndrome (DSS) in India was observed in 1986 in New Delhi [5, 6]. Medicinal plants are a crucial therapeutic aid for various diseases. The plant Leucas is distributed throughout Asia, Africa, and India. It is an erect, stout herb, branched, scaly or pubertal which grows with a height of about 15-60 cm [7]. *Leucas cephalotes* (Roth.) Spreng (family Lamiaceae) is the well-known Ayurvedic traditional medicinal plant used in India to treat several diseases [8]. It is an annual herb, commonly called Dronpushpi (Sanskrit) or Goma in Hindi. The plant is mostly grown as a weed during the rainy season. In India, the decoction of the plant is used orally in the treatment of diarrhoea, fever, jaundice, blood purifier, cold, cough, as an appetizer and emmenagogue. The poultice prepared from the flowers and leaves is applied externally to treat headache [9]. Traditionally, the *L. cephalotes* leaves juice used topically in psoriasis, scorpion sting, skin eruptions, jaundice, asthma, anti-inflammatory, dyspepsia, paralysis and internally for the treatment of urinary complaints [10]. The flower and leaves of *L. cephalotes* also used to cure the parasitic infection, constipation, earache, headache, piles, malaria and migraine. The whole plants are used as insecticides and indicated in traditional medicine for cough, cold, chronic skin eruptions and rheumatism [11]. It is one of the most common historic plants used for the cure of snakebite. *L. cephalotes* have antipyretic action and also considered to be stimulant, expectorant, diaphoretic, anticoagulant, anti-cancerous, antioxidant, anti-inflammatory, anti-diabetic and emmenagogue [12–14]. The plant contains secondary metabolites such as β-sitosterol, triterpenoids, oleanolic acid, ursolic acid, phenolic compounds, diterpenes, alkaloids and glycosides as major chemical constituents [15].

Our health and wealth are highly influenced by various viral diseases like Dengue virus, Chikungunya virus, Herpes Simplex Viruses, Nipah Virus [16], Zika Virus [17], and now COVID-19 [18]. The medicinal plants can play a vital role against these viruses [19–22]. Thus, the researcher’s curiosity in the study of medicinal plants and isolation of secondary metabolites is growing rapidly in recent times. The World Health Organization (WHO) reported that over 80% of the people in emerging countries rely on traditional drugs and about 855 traditional medicines used in the world obtained from crude plant extracts [23]. There are many extraction techniques. In present study Supercritical Fluid Extraction (Applied Separation Inc. U.S.) method was used. SFE is an analytical method which is capable of separating the specific compounds from the unknown mixture of plants at a definite temperature and pressure. Considering *L. cephalotes* antipyretic, antimalarial, and anti-HIV activity, the current study was undertaken to test the anti-dengue activity of *L. cephalotes* supercritical extract and isolated oleanolic acid in vitro and in silico and to identify and characterize the isolated compound by different techniques.

## Methods

### Chemicals and reagents

Various chemicals and reagents were used in the study include 3-(4, 5-dimethylthiazol-2-yl) -2, 5- diphenyltetrazolium bromide (MTT, Hi-Media, batch no.0000263610), phosphate buffer saline (PBS, Hi-Media, batch no. 0000313379), streptomycin sulfate (100 μg/ml, Hi-Media, batch no. 0000187551) and penicillin (100 U/ml, batch no. BCBN 3112 V) antibiotic was purchased from Sigma-Aldrich, USA. Chemical used in cell culture including minimum essential medium (MEM, batch no.0000319279) and trypsin (batch no. 0000285329) were purchased from Hi-Media Laboratories (Mumbai, India). Fetal calf serum (FCS) was purchased from Gibco (NV, USA, batch no.1584260). Chemical including, buffer, enzymes, dNTPs, dengue specific primer, and probes were commercially available in Geno-Sen’s Dengue S1-S4 PCR kit used in the study.

### Plant material collection

*Leucas cephalotes* (Roth.) Spreng whole plant was selected for the present study based on their ethnobotanical uses. A mature and healthy plants were collected in the month of September from the fields of village Majra located at Latitude of 28°41̍ 15.432″ N and Longitude 76°52 ˈ 47.028″ E, District Jhajjar, State Haryana, India where it grows as wild during the rainy season. Identification of the plant was done based on taxonomic keys and comparing with available Herbarium data at M. D University, Rohtak India. The further identification confirmation has been done from Department of Botany of M. D. University, Rohtak, India. The accession number was allotted (MDU 5802, D. DUN 1353). The whole plant parts were washed with tap water to remove the dust and again washed with distilled water and shade dried at room temperature for 20 days.

### Preparation of *L. cephalotes* extracts using supercritical fluid extraction (SFE) machine

Extract of *L. cephalotes* plant was prepared using Supercritical Fluid Extraction Machine (Speed™ SFE Prime of Applied Separation Inc. U.S.). Ten grams of the plant powder was loaded into the stainless steel extraction vessel of the machine. The solvent (CO_2_) flow rates were varied from 1.6 ml/min with static-dynamic mode (1 h static and 30 min dynamic mode). The plant extraction method was standardised and optimised for the isolation of secondary compounds from *L. cephalotes* crude extract (Table 1). The extract was collected in the collection tube and dissolved in double distilled water. The prepared plant extracts were lyophilized (Hyper Cool HC3110, Hanil Scientific Inc.). The dried extracts were weighed and stored at 4 °C for further use. The percentage yields of plant extracts were calculated as follows:- % age yield = Weight of the extract obtained/ weight of dried material× 100.

**Table 1:** Yield obtained from SFE extract of *L. cephalotes*

Temperature	Plant sample	Pressure/Mpa	Yield	% of the yields
40 °C	10 g	10	0.12 g	1.2%
40 °C	10 g	15	0.13 g	1.3%
40 °C	10 g	20	0.10 g	1.0%
40 °C	10 g	25	0.086 g	0.86%

### High-performance thin layer chromatography (HPTLC) analysis

The marker oleanolic acid (> 97%) was purchased from Sigma Aldrich (India) and the compound was dissolved in HPTLC grade methanol in various aliquots (2 μl, 4 μl, 6 μl, 8 μl and 10 μl). *L. cephalotes* extract stock (10 mg extract/ml methanol) was prepared for HPTLC. The dissolved test samples were filtered using a 0.45 μm membrane filter (MILLEX® GV) before applying to the silica gel plates. The stocks solution was preserved at − 20 °C till further use.

### HPTLC analysis and compound isolation

The HPTLC analysis was carried out by using Lino mat V applicator, TLC scanner 3, twin trough plate development chamber and win CATS 3 software 1.4.8 (Switzerland). For oleanolic acid isolation, the mobile phase was used as Toluene: ethyl acetate: formic acid (80,20:0.1 v/v) [24]. The TLC silica gel plate (10 × 10 cm, 60 F254, E. Merck) was pre-washed with methanol and activated at 100 °C for 10 min. The different concentration of the standard oleanolic acid was applied on TLC plates, 10 mm above from the bottom by using CAMAG automatic sample applicator (Lino mat V) with N_2_ flow. 5 μl of plant supercritical extract was applied on the TLC plate in duplicate by using a Hamilton microsyringe (100 μl) in the form of 5 mm wide bands. The plate put into a twin trough developing glass chamber and pre-saturated with 20 mL of the solvent system (mobile phase). The chromatogram of each spot was developed up to 8 cm in height of the plate and dried at room temperature/CAMAG, TLC plate heater at 100 °C for 10 min. The plate was photo-documented in visible, UV light in between the range of 200–600 nm. The resolved spot was used to determine the retention factor (R_f_) value and compared it with the R_f_ value of the marker i.e. oleanolic acid. The preparative TLC gel plate was used for the isolation of oleanolic acid. The band of oleanolic acid was identified by comparing it with the marker oleanolic acid band. The band was scratched and dissolved in 1 mL double distilled water. The silica was removed by centrifugation and then oleanolic acid (OL) was collected into 2 ml vials and lyophilized.

### Identification and characterization of the isolated compound from the SFE extract of *L. cephalotes*

The identification of the isolated compound was elucidated with the help of FT-IR, UV-Vis spectrophotometer, HPTLC along-with marker compound, Differential scanning calorimetry (DSC), and Proton and Carbon Nuclear magnetic resonance (NMR).

### Fourier transform infra-red spectroscopy (FT-IR)

The marker oleanolic acid and the isolated compound from the supercritical extract of *L. cephalotes* was examined by FT-IR spectroscopy (Bruker, Germany) for the detection of different functional groups and molecular structures in the wave range of 400–4000 cm^− 1^ at a resolution of 4 cm^− 1^.

### Ultraviolet-visible spectroscopy (UV-vis)

The characterization technique was also performed by UV-Vis spectroscopy using Shimadzu UV-2450 spectrophotometer, Japan. The wavelength range for absorption was 200–600 nm. The double distilled water was used as a blank.

### Proton nuclear magnetic resonance spectroscopy (^1^HNMR and ^13^CNMR)

^1^HNMR and ^13^CNMR spectra were run on Bruker Avance III, 400 Mhz (Agilent, USA) in Cdcl3. Chemical shifts are reported as values, in ppm and tetramethylsilane (TMS) used as an internal standard in the NMR spectrum.

### C6/36 cells culture

The C6/36 Aedes cell line (ATCC® CRL-1660 TM) was maintained in minimum essential medium (MEM) supplemented with 10% fetal calf serum (FCS), 2mML- glutamine, penicillin (100 U/ml) & streptomycin (100 μg/ml). Cultured cells were incubated at 28 °C in a humidified atmosphere, with 5% CO_2_ [25, 26]. The medium was changed twice a week.

### A stock preparation of *L. cephalotes* for cell viability and antiviral assays

3000 μg *L. cephalotes* SFE extract and the 3000 μg isolated compound were weighed and dissolved in 1 ml of minimum essential medium (MEM) on the basis of the solubility. The pH of the medium was maintained at 7.0. The plant extract and isolated oleanolic acid were diluted to varying concentration (1500 μg/ml to 23.43 μg/ml) in the 96 well plates. The dissolved extract was filtered using a 0.22 μm syringe filter (MILLEX® GV). Extracted stocks were preserved at − 20 °C for further use.

### Estimation of maximum non-toxic dose (MNTD) by MTT in vitro

For cell viability evaluation of plant SFE extracts or oleanolic acid, the C6/36 cells (1.2 × 10^6^ cells/well) were seeded into 96-well flat-bottom plates (Nunc, Thermo Fisher Scientific, USA) and incubated overnight at 28 °C in a 5% CO_2_ incubator. Briefly, 80% confluent cells were treated with different concentrations (1500 to 23.43 μg/ml) of *L.cephalotes* extract and isolated compound in triplicates. A cell control (Cells with medium) without any test sample and blank control (medium) were also plated. After incubation of 96 h, the medium was discarded and replaced with 20 μL of 3-(4, 5- dimethylthiazol-2-yl) 2,5-diphenyl tetrazolium bromide salt solution (MTT, 5 mg/1 ml in PBS) and incubated for 3 to 4 h at 28 °C in 5% CO_2_ incubator. After that, the solution of each well was discarded without disturbing cells. Then 100 μl of DMSO (Sigma Aldrich, USA) was added into each well to stop the reaction followed by continuous shaking for 15 min till all the formazan crystals were dissolved. Afterwards, absorbance values were noted by using a microplate reader (Bio-Rad, USA) at 595 nm. The percentage of viable cells of *L. cephalotes* and oleanolic acid were determined in relation to control cells using mean values of the triplicate experiment. Similarly, maximum non toxic dose for toxicity assay of SFE extract was analysed in mammalian cell lines (Vero cell lines).

### Virus culture

A total of 500 μl of an appropriate dilution of dengue-2 standard strain was inoculated onto confluent C6/36 cells in a T-25 cm^2^ tissue culture flask. The inoculum was incubated at 28 °C in 5% CO_2_, shaking every 5 min to maximise the viral adsorption to the cells. After 55 min, the virus growth medium (VGM) was added to the flask and the cells flask were further incubated for 9 to10 days and observed daily under an inverted (Magnus, India) microscope for the presence of any possible cytopathic effect. These were repeated various times until sufficient virus stock was collected. Even after proper incubation, the cytopathic effect was not seen. The lysates were harvested and stored in a deep freezer (− 80 °C). Direct methods of quantitation like TCID_50_ or plaque assays are not conveniently for the dengue virus since they do not generate any morphological changes in the cells [27]. Viral RNAs of lysate was extracted by using a commercial QIAmp Viral RNA mini kit (Qiagen, Germany) according to the manufacturer̕ s protocol. The viral titer (copy number) of the dengue-2 virus was determined in culture lysate by real-time amplification by using commercial quantitative Geno-Sen’s dengue 1–4 kit which contains known standards of dengue (10^1^, 10^2^, 10^3^, 10^4^ and 10^5^ copies/μl). Hundred copies/ml was used in the further antiviral experiment.

### In vitro anti-dengue assay

The anti-viral assay was performed in a 96 well culture plate with monolayers of the C6/36 cell line. The assay was performed in 96 well plates with the controls; which included the cells only (negative control), a dengue-2 virus (TR-1751) control which contain 10, 100, 1000 viral copies/ml in duplicates as a positive control. The experiment was carried out by mixing the 100 copis of the virus suspension. Virus suspension was pre-treated with non-toxic concentration of plant extract (*Leucas cephalotes*) / test compound (oleanolic acid) for 55 min with the gentle shaking after every 10 min in replica plates. The pretreated virus were transferred to respective wells of C6/36 cell lines and incubated for 55 min., shaking gently every 10 min. The medium was aspirated from the wells after the inoculums had adsorption. Then 100 μl virus growth medium was added without disturbing the cells layer. Further, the culture plates were incubated at 28 °C in a CO_2_ incubator for 7 days without disturbing the cells. The plate was frozen at − 70 °C after proper incubation, and the lysates were harvested and stocked into 2 ml vials. Following that, RNA was extracted from each vial [28, 29]. Further, the antiviral effect of plant extract and isolated test compound were determined by using real-time RT- PCR against dengue-2 serotype with the positive control.

### RNA extraction

Viral RNA was isolated from 140 μl of culture supernatant using a commercial RNA mini kit (QIAmp Viral RNA kit, Qiagen, Germany) according to the manufacturer ʼ s protocol. Final elution was done in 50 μl buffer before storing at − 80 °C until use for anti-viral assay.

### Quantitative anti-viral assay

Real-time RT- PCR was performed to determine the inhibitory effect of plant extract or its compound oleanolic acid on the DENV-2 serotype. The experiment was done by using the commercially available quantitative kit (Geno-Sen’s Dengue 1–4 PCR kit). The quantitation standards provided in the kit dengue 1–4 serotypes (10^1^–10^5^ copies/μl) are treated in the same way as extracted samples and the same volume is used i.e. (15 μl). The kit contains a specific master mix (buffer, enzymes, dNTPs, dengue specific primer, and probes) for specific amplification and quantification of dengue viruses. The RNA was prior extracted from each lysate (sample). The experiment was performed in an ABI 7500 real-time PCR instruments. Before starting, all the PCR reagents were thawed and mixed properly. The master mix was prepared following the kit manufacturer’s instructions. After that, the desired number of PCR tubes were prepared by adding 10 μl master mix and 15 μl of extracted RNA to each lysate tube along with 15 μl of the standards (Dengue 1–4, S 1–5) must be used as a positive control and 15 μl of water (Water, PCR grade) as a negative control. Then, all the reagents in the PCR tubes were mixed properly by pipetting up and down. The PCR tubes were closed and transferred into the ABI 7500 in real time. The thermocycler amplification conditions were as follows: reverse transcription at 50 °C for 15 min, denaturation at 95 °C for 10 min, as followed by 45 cycles of denaturation at 95 °C for 15 s, annealing at 55 °C for 30 s and a final extension step at 72 °C for 15 s. The fluorescence emission data were collected during the annealing step. The standard curve or amplification curve was generated as above can also be used for quantitation in subsequent runs, provided that at least one/two standard is used in the current run with back titration.

### Data analysis

The percentages of cell viability of the *L.cephalotes* SFE extract or oleanolic acid were analysed by Microsoft Excel 2007 with the help of Tukey’s test (each treatment mean value different from each-others and compared to positive control). Samples were assayed in triplicates. The results were expressed as the average value of all wells and calculated the cell viability of plant and test compound by using this formula:-.

$$ \mathrm{Cell}\ \mathrm{viability}\ \left(\%\right)=\frac{\mathrm{Absorbance}\ \mathrm{treated}\ \mathrm{cell}\hbox{-} \mathrm{Absorbance}\ \mathrm{blank}}{\mathrm{Absorbance}\ \mathrm{cell}\mathrm{s}\ \mathrm{control}\hbox{-} \mathrm{Absorbance}\ \mathrm{blank}}\times 100 $$

### Preparation of ligand and protein structure

The 3D structures of plant ligand i.e. oleanolic acid (PubChem IDs: 10494) was downloaded from PubChem. Structures were then minimized and prepared for docking using dockprep module of the chimera. The 3D structures of dengue-2 viral non-structural proteins NS1 (PDB IDs: 4O6B) and NS5 (PDB IDs: 4V0Q) were downloaded from PDB (protein data bank). Bound prosthetic groups like NAG, Zn, Acetate ion, Glycerol were removed and protein structures were minimized using chimera. Auto dock software (V4.2.6) was used for docking analysis between dengue NS1 and NS5 protein and selected ligand oleanolic acid. For docking analysis protein was prepared by merging polar hydrogen with carbon and addition of Kollman charges. The Grid box was extended to the whole protein to perform blind docking with a spacing of 0.375 Å. The ligand was prepared by managing total torsions available making the ligand a bit flexible molecule. The search algorithm used for docking was a Lamarckian Genetic Algorithm (4.2). The docking complex was saved using the MGL tool and interaction was visualized by Lig Plot plus.

Molecular Docking of NS1 and NS5 against their known natural receptors Lectin and Valporic acid respectively alongwith ligand (Oleanolic acid) was also done using CCDC GOLD (Genetic Optimization for Ligand Docking) as described earlier (30). It was done to validate the docking tool, and compare the binding of NS1 and NS5 proteins. Docking was performed with hundred genetic algorithms (GA) run for each compound. In a single GA run, 1,00,000 operations were performed on a population size of 100 individuals with a selection pressure of 1.1. The number of islands was set to 5 with a niche size of 2. The values for crossover, mutation and migration were set as 95, 95 and 10 respectively.

## Results

### Optimum extraction condition

Supercritical fluid extraction (SFE) is an advanced analytical technique that is capable of separating the different compounds from the botanical plant materials when applied to different parameters. SFE extraction condition was optimized at 40 °C temperature and 15Mpa pressure for isolation of secondary compounds from the crude extract of *L. cephalotes*. The maximum yield of the extract was obtained 0.13 g/10 g (1.3% w/w) (Table 1). SFE is a safer, time saving, separating the compound from the non-flammable solvent (CO_2_). The properties of SFE can be altered by changing the temperature and pressure for selective extraction.

### Maximum non-toxic dose of plant extract

**Fig. 1:**
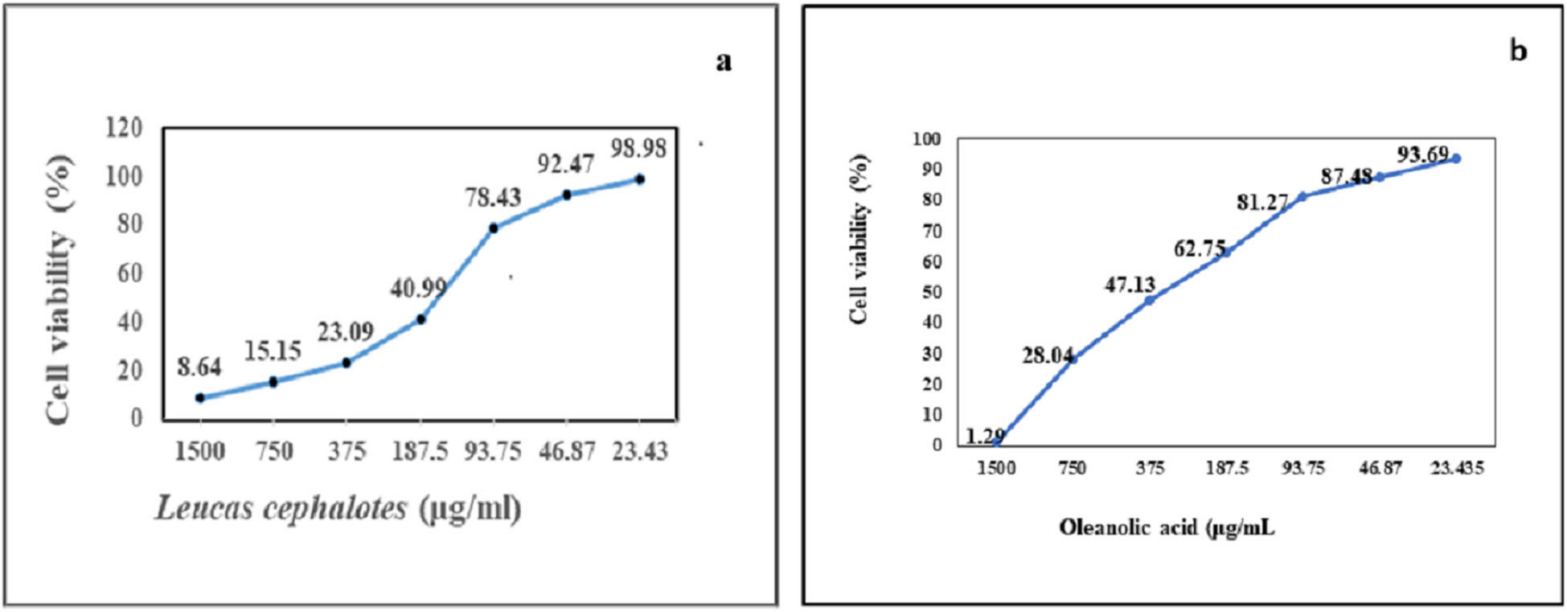
**a**) Maximum non-toxic dose (MNTD) of *L. cephalotes* SFE extract **b**) MNTD of oleanolic acid

Cell viability/toxicity was evaluated by MTT assay in the C6/36 cell line and the maximum non-toxic dose of *L. cephalotes* SFE extract and isolated oleanolic acid were calculated as 46.87 μg/ml (Fig. 1a) and 93.75 μg/ml (Fig. 1b), respectively. Likewise, cell viability/toxicity evaluated by MTT assay in the vero cell line of plant extract was noted 46.87 μg/ml.

### Quantitative antiviral assay by real-time PCR assay

**Fig. 2:**
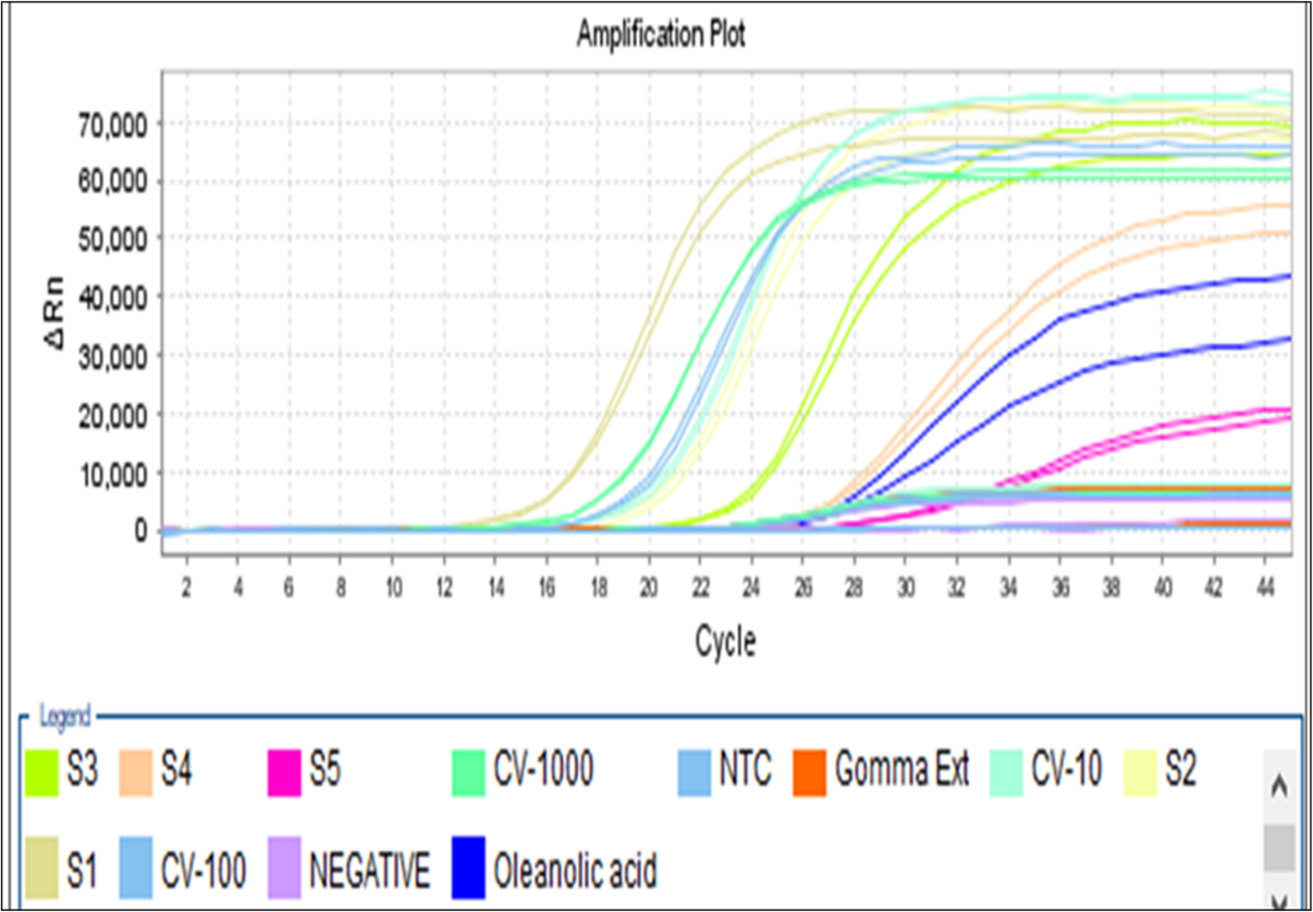
Representation of dengue viral inhibition by *L. cephalotes* SFE extract and oleanolic acid standard curve (a) and amplification curve (b): (S1 to S5 are positive vius control; CV-10, CV-100 and CV-1000 copies/ml; Gomma (*L. cephalotes* extract); Blue- oleanolic acid curve

This study revealed that the *L. cephalotes* supercritical extract and isolated oleanolic acid showed 100% inhibition and 99.17% against the dengue − 2 virus treated with a concentration of 46.87 μg/ml and 93.75 μg/ml, respectively. The anti-dengue activity of isolated oleanolic acid compound carried out by real-time RT-PCR has been given in Table 2. The amplification curve depicting the anti-dengue activity of plant extract and oleanolic acid are shown in Fig. 2.

**Table 2:** The anti-dengue activity of isolated oleanolic acid

Serial no.	Compounds name	Quantity Mean values (Virus copies/ml)	Quantity (SD value)	CT Mean value	CT (SD Value)	^a^% inhibition of compound against dengue virus
1	Oleanolic acid	23,307.19	2527.09	26.31	0.20	99.17%
2.	Virus positive control (100 copies/ml)	2,808,999.0	102,602.78	17.68	0.07	–

^a^% of inhibition = Mean value of test sample/average of virus control cells) × 100

### Fourier-transform infrared spectroscopy (FT-IR)

**Fig. 3:**
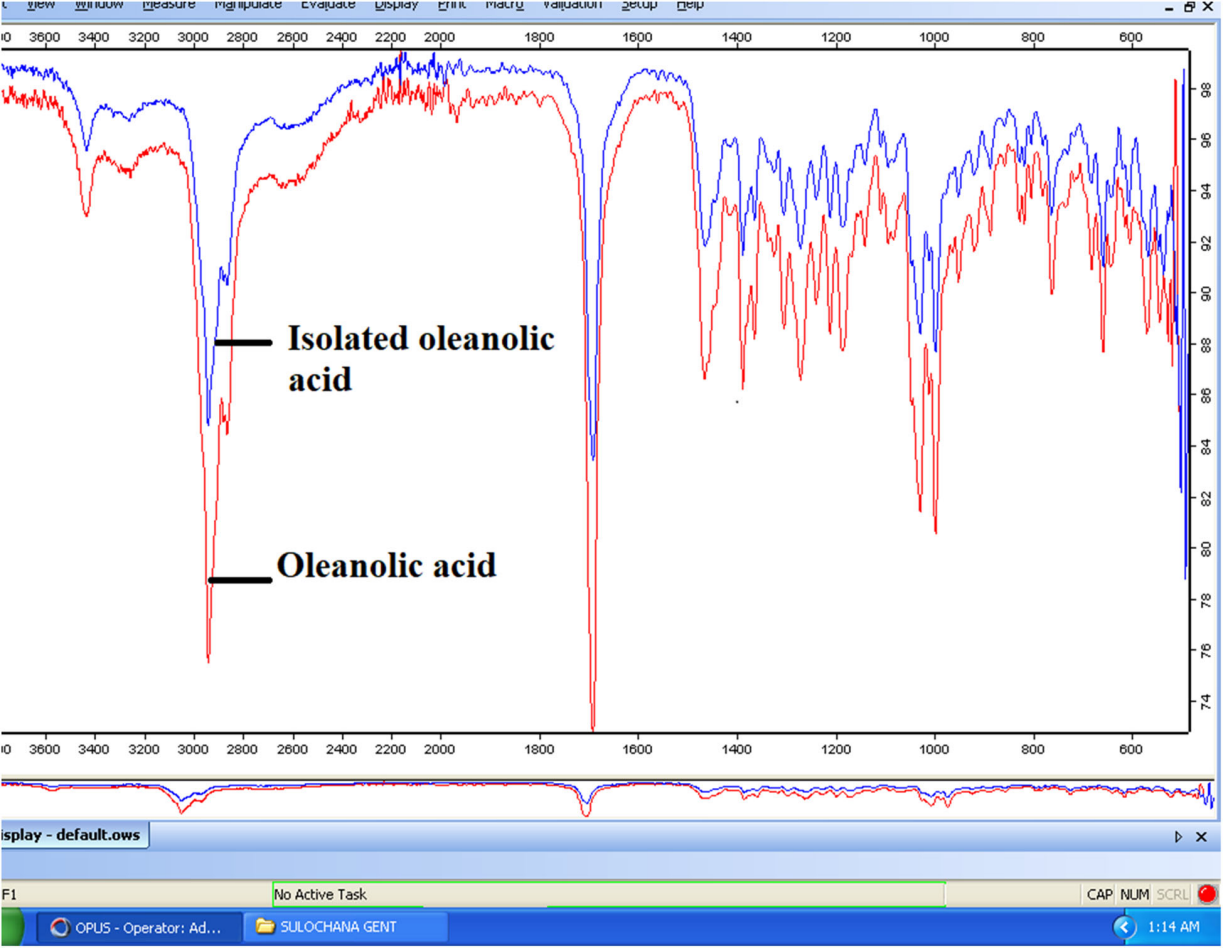
Superimposed FT-IR image of isolated oleanolic acid with marker

The FT-IR spectrum of *L. cephalotes* crude extract showed the prominent main transmittance bands at 3436, 2939, 2836, 1690, 1463, 1387, 1363, 1303, 1270, 1209, 1185, 1139, 1092, 1027,1009, 994, 996, 917, 884, 826, 816, 760, 679, 656 and 573 cm^− 1^. The peak at 3436 cm^− 1^ (O-H stretching) suggesting the presence of alcohol and a carboxylic acid group. The band at 3000–2800 cm^− 1^ (−C-H-asymmetric & symmetric stretching) showed the presence of alkanes. The spectra at 2000–1650 cm^− 1^ (strong -C=O- stretching) showed the presence of an unsaturated aldehyde. FT-IR spectra at 2918–2389 cm^− 1^ and 1650–1580 cm^− 1^ (−C-H-bending) showed the presence the alkane compounds and at 1420–1330 cm^− 1^ (−O-H-stretching) suggested the presence of alcohol and at 1342–1266 cm^− 1^ (−C-N stretching) suggested the aromatic amines. The peak at 1275–1020 (−C=O- stretching) showed alkyl aryl ether and the peak at 1210–1020 (−C=O- stretching) suggested the presence of aliphatic ether and alkyl ether and at 995–885 (Strong C=C bending) showed the presence of alkenes group and 850–550 cm^− 1^ (Strong C-Cl stretching) were suggested the halo-compounds. The curves at 3433, 2939, 2871, 1690, 1500, 1463, 1387, 1250, 1209, 1137 and 656 cm^− 1^ were found similar in the plant SFE extract and oleanolic acid (marker compound) (Fig. 3).

### Ultraviolet-visible spectroscopy analysis

**Fig. 4:**
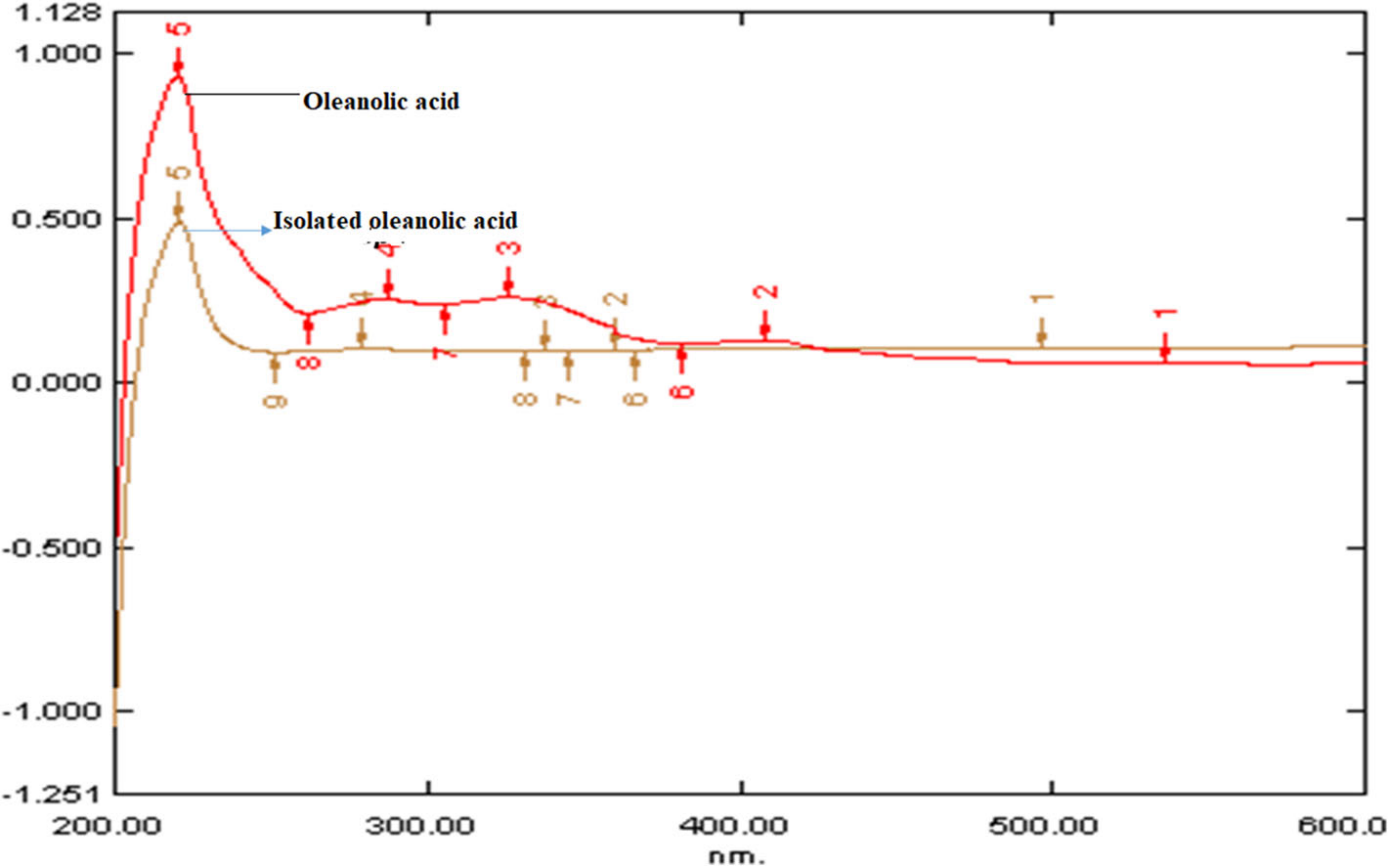
UV overlay spectra of isolated oleanolic acid with marker

The overlay spectra of oleanolic acid and *L. cephalotes* extract was obtained between 210 to 240 nm. Broadband was observed at 232 nm in the oleanolic acid biomarker. This indicates that the compound oleanolic acid was present in the plant extract and the isolated compound has found similar properties to standard oleanolic acid spectra (Fig. 4). The melting and boiling point of oleanolic acid is > 300 °C and 553.00 to 554.00 °C, respectively. The IR and UV spectra of the isolated sample and the standard were superimposable.

### HPTLC analysis

**Fig. 5:**
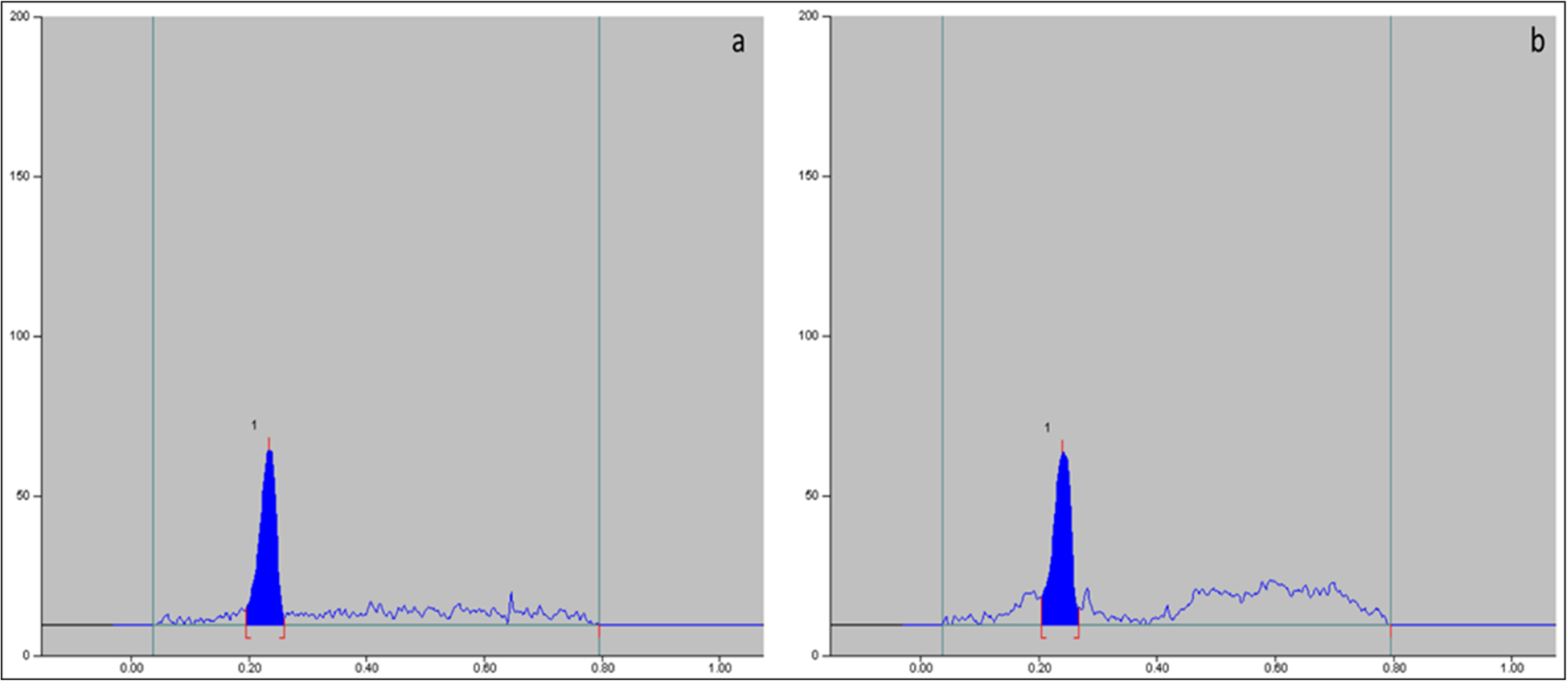
**a**) *L. cephalotes* SFE extract densitogram **b**) HPTLC densitogram of marker oleanolic acid

The result of HPTLC analysis confirmed the presence of oleanolic acid (OL) in plant extract (Fig. 5a) compared with the marker compound (Fig. 5b) chromatogram. The plant extract has indicated the same R_f_ value to the marker compound (0.19 ± 0.06). TLC of plant extract with marker compound is depicted in Supplementary Fig. S1. The purity of oleanolic acid was established by HPTLC analysis of the isolated compound that shows a single peak and UV absorption spectrum completely overlapped with an absorption maxima at 210 to 240 nm. The purity of the compound was found 98.27% with a melting point of 311.16 °C as revealed by DSC spectra (see Supplementary Fig. S2). The linearity equation of oleanolic acid was generated by regression analysis and the data showed a good linear relationship over a concentration range of 2.0–10.0 μg/spot (see Supplementary Fig. S3). This R^2^ value signifies how close the data fit the regression line.

### Estimation of oleanolic acid in the SFE extract

The amount of oleanolic acid present in the extract was calculated by the linear regression lines (Y = 129.03x + 258.52). The oleanolic acid quantity was detected 33.06 μg/ml in the 50 μg crude extract of *L. cephalotes*. The average recovery of oleanolic acid content in the supercritical extract from *L. cephalotes* was found to be 66.12% w/w.

### Proton and carbon nuclear magnetic resonance (NMR)

**Fig. 6:**
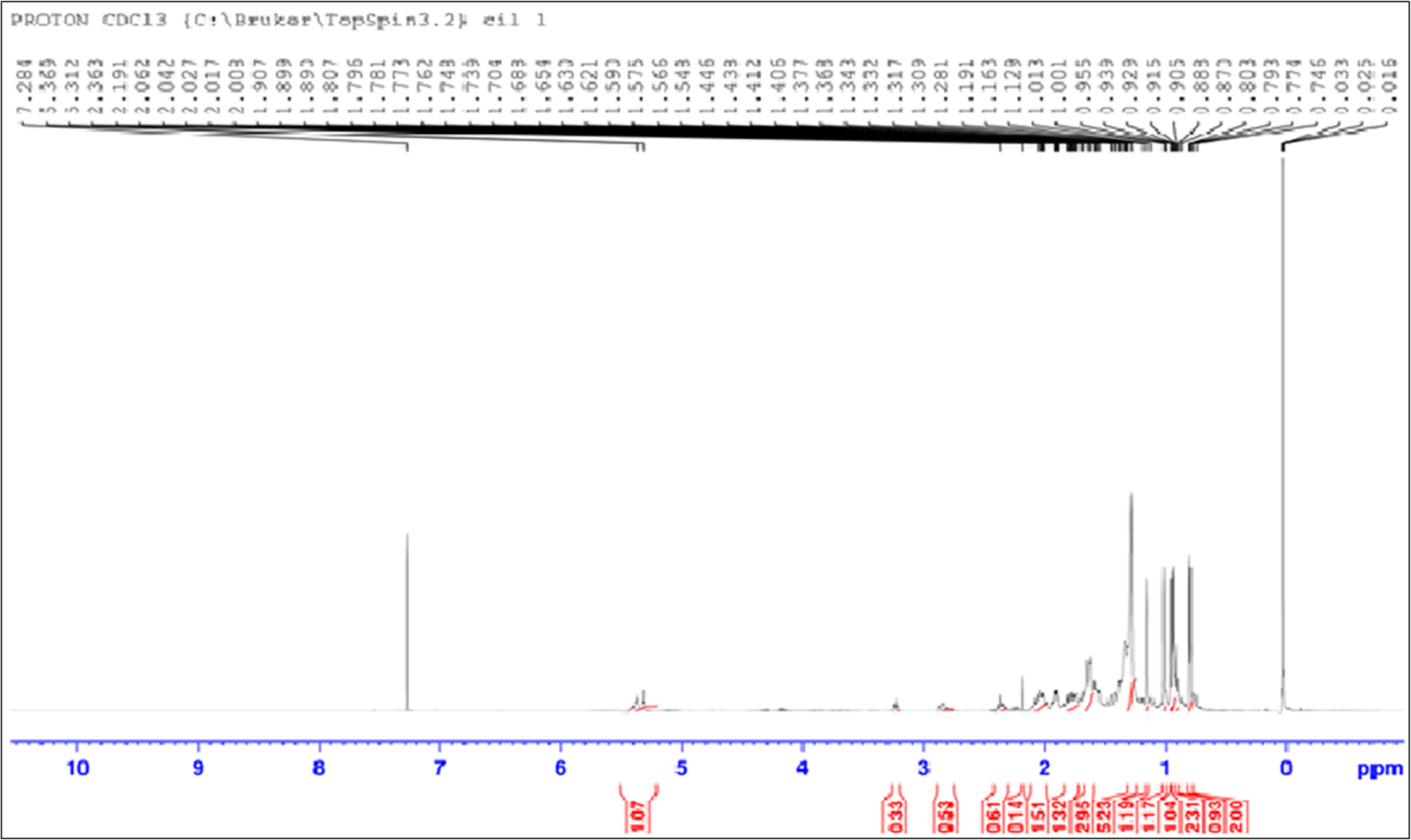
^1^HNMR spectra of isolated oleanolic acid

**Fig. 7:**
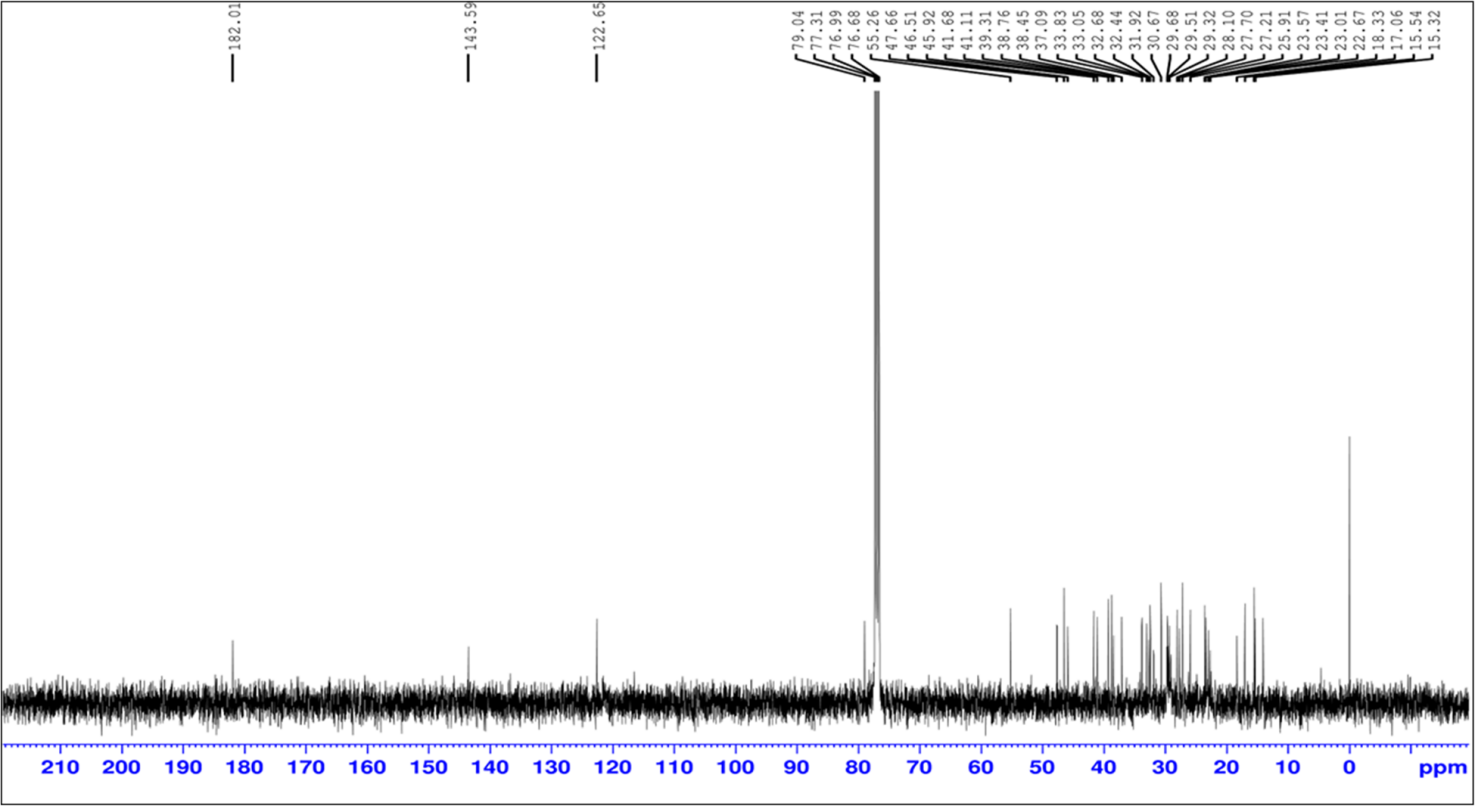
^13^CNMR of isolated oleanolic acid

The Proton ^1^HNMR analysis results are resembles to oleanolic acid structure 1.129(δ, 12H, 4CH3), 1.228 (m, 4H, CH2 C6H5) 1.191(δ, 3H, CH3), 1.37(S, SH, CH3), 1.78 (m, 4H, CH2, C6H5), 1.16 (s, 6H, CH3), 1.57 (m, 4G, CH21, C6H5) (Fig. 6).^13^CNMR: 23.41, 25.91, 27.21, 27.70, (4C, 4CH3), 182.01(1C, COOH), 30.67, 31, 33.68, 37.09, 38.76 (6C, C6H6), 55.26, 122.65, 143.59, 76.68, 6C, C6H3), 31.92, 32.44, 33.83, 41.11 (4C, C6H5) (Fig. 7). As a result, we can conclude that our prepared plant extract is matched to the oleanolic acid structure (see Supplementary Fig. S4). Oleanolic acid (3*β*-hydroxyolean-12-en-28-oic acid) is a pentacyclic triterpenoid with widespread occurrence throughout the plant kingdom.

### Molecular docking with dengue non-structural proteins (NS1 and NS5)

**Fig. 8:**
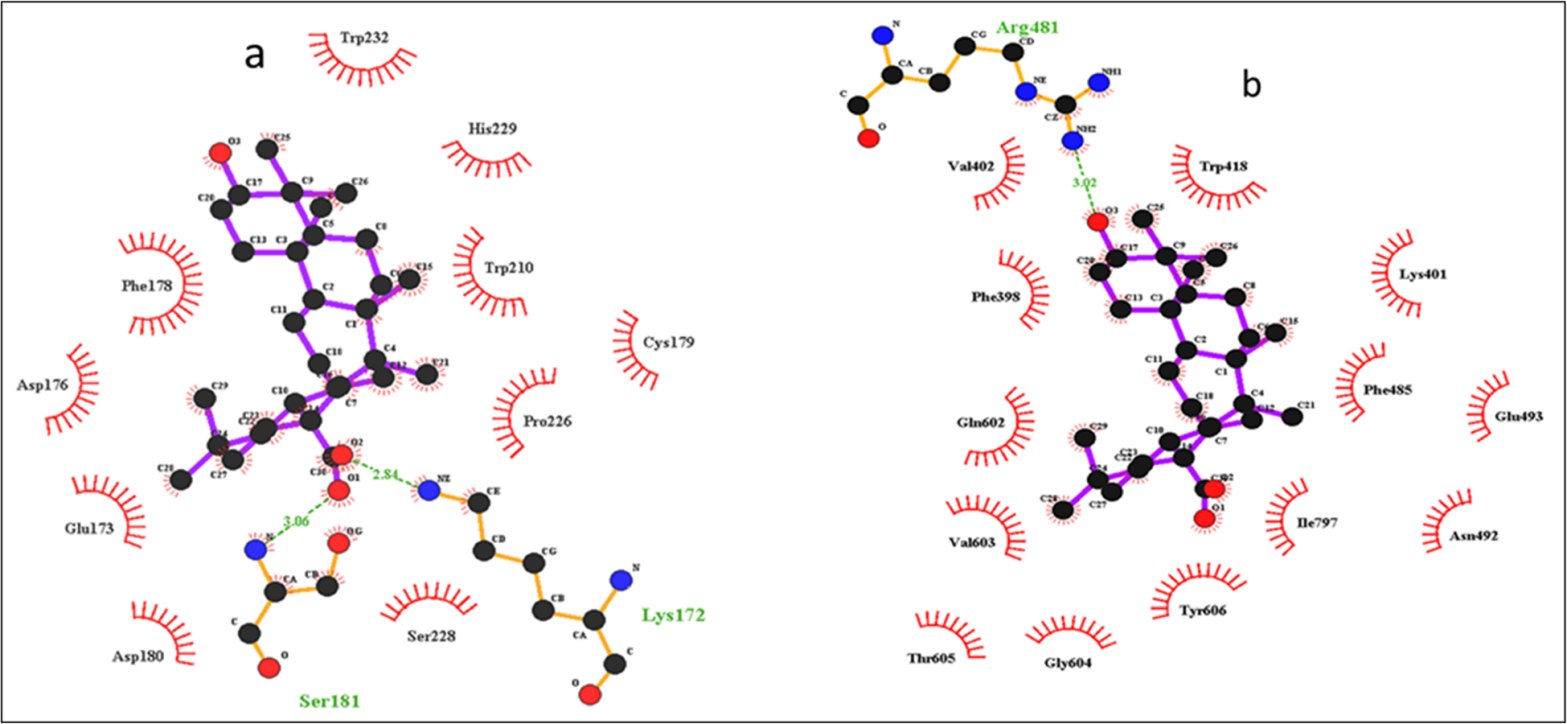
**a**) Ligplot showing the amino acids involved in interactions with oleanolic acid with NS1 protein, **b)** Ligplot showing the amino acids involved in interactions with oleanolic acid with NS5 protein

**Fig. 9:**
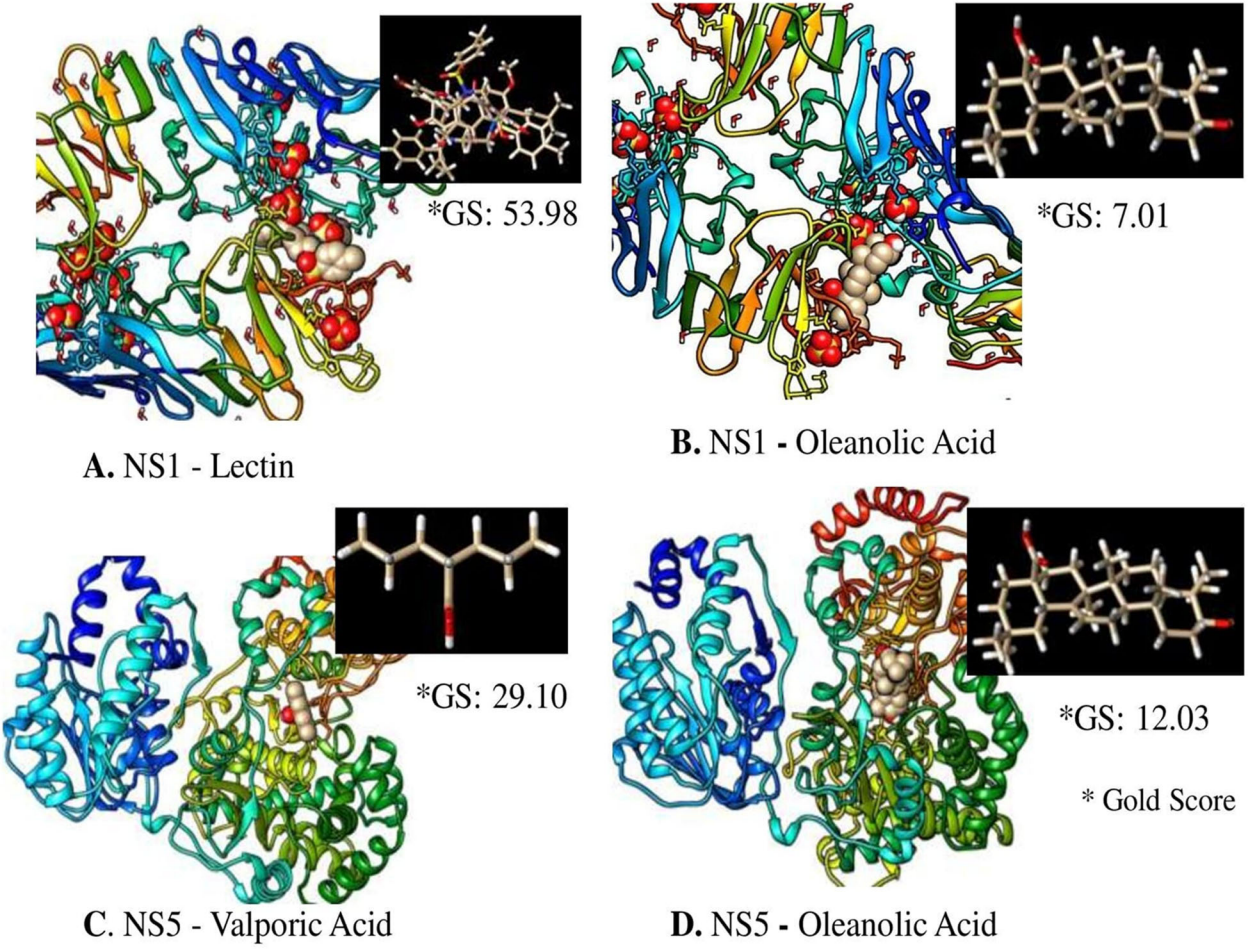
Molecular docking of A. NS1 with Lectin; B. NS1 with Oleanolic acid; C. NS5 with Valporic acid and D. NS5 with Oleanolic acid using CCDC GOLD software

Oleanolic acid shows the maximum binding with both dengue virus proteins; NS1 and NS5 (− 9.42 Kcal/mol and − 8.32Kcal/mol) respectively. Further analysis was done on the basis of H-bond and interacting residues. Oleanolic acid showed 2 interactions against protein NS1 with binding energy of − 9.42 Kcal/mol and Lys 171 and Ser181 as the interacting residue and H-bond distances of 2.84 Å and 3.06 Å and nearby interacting residue Trp232, Phe178, Asp176, Glu173, Asp180, Ser228, Pro226, Cys179, Trp210, His229 (Fig. 8a) while oleanolic acid showed a single interaction against protein NS5 with binding energy − 8.32Kcal/mol and Arg481 as the interacting residue and the H-bond distance of 3.02 Å and nearby interacting residue Val402, Phe398, Gln602, Val603, Thr605, Gly604, Tyr606, Ile797, Phe485, Asn492, Glu493, Lys401, trp418 (Fig. 8b). The docking scoring function of oleanolic acid was 125.11 nano-molar for NS1 protein while for NS5 protein it was 798.18 nano-molar. The inhibition and electrostatic values were found as + 798 μM and − 0.21Kcal/mol. The van der Waals and hydrogen bond energy was found − 9.01Kcal/mol. Molecular docking showed the maximum Gold score of 53.98 and 29.10 with NS1-Lectin and NS5-Valporic acid natural receptors respectively; whereas, NS1 and NS5 revealed the Gold score of 7.01 and 12.03 with the ligand oleanolic acid (Fig. 9).

## Discussion

Dengue and others viruses are still a challenge for the world and have become a global health problem. World Health Organization has deep concern over dengue because a large part of the world population is affected. Medicinal plant and their derivatives play an important role to combat against dengue virus. Various type of drugs derived from medicinal plants i.e. morphine, quinine, quinidine, artemisinine, taxol, aspirin, colchicines, digoxin, tubocurarine, ephedrine, vincristine and vinblastine are available in the market to fight against many types of disease. Anti-dengue virucidal activity of methanol extracts of *Andrographis paniculata*; essential oils of *Santalum album*; petroleum ether extract of *Alternanthera philoxeroides*; ethanol and water extracts of *Hippophae rhamnoides;* dichloromethane and ethanol extracts of *Cladogynos orientalis, Rhizophora apiculata, Flagellaria indica, Houttuynia cordata,* and methanolic seed extracts of *Quercus lusitanica* have already been reported [22, 30]. In quantitative term few plants showed anti-dengue activity viz., Kaushik et al. study demonstrated that andrographolide extracted from *A. paniculata* showed 97.23% anti-dengue activity against the dengue-2 virus in C6/36 cell lines [31]. *Cyamopsis tetragonoloba* SFE extract showed the 99.9% inhibition against the dengue-2 virus [32]. In the present study *L. cephalotes* SFE extract inhibit 100% and test compound inhibit 99.17% dengue-2 virus in C6/36 cells line.

The antiviral studies have not been performed on *Leucas cephalotes* plant and its SFE extract. In the present work we have chosen SFE extraction method. None of study was reported on SFE extract of *L. cephalotes.* The optimum yield of the extract was obtained at 40 °C temperature and 15Mpa pressure. Sofi et al. study reported 11% w/w yield of *L. cephalotes* by Soxhlet method [33]. Rahman et al., reported 3.16% yield in ethanolic extract of *L. cephalotes* leaves by cold extraction method [34]. In present study SFE extraction yield was 1.3%. The SFE extract were found to contain oleanolic acid. The present work provides us with a cost-effective, reliable and safer option to treat dengue virus infection. The SFE extract of *L. cephalotes* found rich in oleanolic acid. Oleanolic acid and its derivatives have been reported for various types of pharmacological activities, such as anti-inflammatory, antioxidant, anticancer, hepatoprotective, weak anti-HIV and weak anti-HCV activity [35–37]. Triterpenoids have been reported to possess antioxidant properties since they prevent lipid peroxidation and suppress superoxide anion generation. The oleanolic acid was characterised by its spectral data (UV, FT-IR, TLC, NMR, DSC) and found that it matched well with that of standard oleanolic acid. The triterpenes have a history of medicinal use in many Asian countries. Like oleanolic acid, the other phytochemicals such as quercetin, sulfated galactomannans, 7-O-methyl-glabranine, flavonoids, glabranine, eugenol, ursolic acid, azadirachtin, D-galactose, carrageenan, chalcone 4- hydroxypanduratin A and panduratin isolated from different plants have been reported for anti-dengue virucidal activity [22, 30]. Some already reported compounds possess anti-dengue activities are given in Table 3 [38–41]. The oleanolic acid isolated in the present study revealed the 99.17% activity against dengue-2 virus while the SFE extract revealed the 100% anti-dengue activity. The differences in the antidegue activity of plant SFE extarct and oleanolic acid may be due to the presence of some other metabolites present in minor quantity of SFE extract of *L. cephalotes*. Researchers are particularly interested in discovering natural compounds that can be used as anti-dengue medicines [42].

**Table 3:** Anti-dengue effects of potent natural products/extracts

Natural products/Extracts	Virus strain	Culture	Proposed mechanism	References
Aqueous leaf extract (*Azidarachta indica*)	DENV-2	C6/36 cells	Undefined	[37]
Petroleum ether, ethyl acetate, ethyl ether and coumane (*Alternanthera philoxeroides*)	DENV	C6/36 cells	Undefined	[37]
Flavonoids and cyclohexenyl (*Boesenbergia rotunda*)	DENV-2	C6/36 cells	Inhibition of dengue-2 virus NS3 protease	[37]
Narasin	DENV-2	Huh-7 cells	Disrupts viral protein synthesis	[37]
Quercetin	DENV-2	C6/36 cells	Inhibits viral replication	[37, 38]
Polyphenol (*Sambucus nigra*)	DENV-2	BHK-21, VERO cells	Undefined	[37]
Ethanol extract of leaves (*Senna angustifolia, Tridax procumbers*), and methanol extract of leaves (*Vernonia cinerea*)	DENV-2	VERO cells	Undefined	[37]
Baicalein	DENV-2	VERO cells	Virucidal activity against extracellular virus	[37]
Chebulagic acid and punicalagin (*Terminalia chebula*)	DENV-2	VERO cells	Inactivate free virus particles and inhibit early viral entry	[37]
Schisandrin (*Schisandra chinensis*)	DENV	VERO cells	Inhibits DENV replication	[37]
4-hydroxypanduratin A	DENV-2	–	Virucidal activity	[22, 39]
Ursolic acid	DENV-2	Huh-7, BHK-21, A549 HEK-293 T	Virucidal activity	[40]
luteolin	DENV-2	Huh-7, BHK-21, A549 HEK-293 T	Virucidal activity	[40]
Indirubin	DENV-2	Huh-7, BHK-21, A549 HEK-293 T	Virucidal activity	[40]
Apigenin	DENV-2	Huh-7, BHK-21, A549 HEK-293 T	Virucidal activity	[40]
Esculetin	DENV-2	Huh-7, BHK-21, A549 HEK-293 T	Virucidal activity	[40]
Oleanolic acid	DENV-2	C6/36	Virucidal activity	Present study

The molecular docking in silico method is used in drug development. In this method, phytochemicals are matched with viral targets to find interactions between the drug and disease-producing agents by using the computational method. The RNA genome of the dengue virus encodes 7 nonstructural proteins that are essential for viral replication (NS1, NS2A, NS2B, NS3, NS4A, NS4B and NS5). NS1 and NS5 are essential for viral replication. NS1 is the only protein that is continuously secreted by infected host cells. The pathogenic roles of NS1 are vascular leakage and severe dengue by disrupting coagulation. NS5 is the largest protein found in the genome of flavivirus. NS1 detected very earlier stage during the infection of the dengue virus. NS5 is Flavivirus’s largest and most drug-targeted region, and it contains methyltransferase and RNA-dependent RNA polymerase (RdRp). NS5 down-regulates the host immune interferon response and modulating RNA splicing at the 5’UTR within the host cell [43, 44]. Excellent drug targets can be identified with the help of bioinformatics. The researchers have put great effort in search of effective molecules for targeting different structural or non-structural proteins of the dengue virus. In the present study oleanolic acid shows maximum binding with both dengue virus protein NS1 and NS5 (− 9.42Kcal/mol and − 8.32Kcal/mol), respectively. In the molecular docking study, the least binding energy revealed the stronger docking between ligands and viral targets. The inhibitory mechanisms of SFE extract of *L. cephalotes* and oleanolic acid can be used to manage dengue. Two possible mechanistic pathways could be the mode of antiviral action i.e. interference with viral adsorption on the target cells and inhibition of virus replication [31, 45]. The present study revealed that *L. cephalotes* extract and its compound showed virucidal activity against dengue virus, DENV-2 strain. It may be due to the presence of oleanolic acid in the plant extract that inactivates certain important structural and non-structural protein of the dengue virus and the enzymes involved in the replication. The molecular docking results also showed that the non-structural proteins; NS1 and NS5 are potential targets of inhibition. Further, molecular docking of NS1 and NS5 with standard known ligands, like lectin and valporic acid (VPA) gave good Gold score and comparable to our test molecule oleanolic acid. This indicates that oleanolic acid can be used as a new molecule to block the binding site of dengue virus non-structural proteins. Earlier, it has been reported that NS1 competitively bind to lectin and neutralize the viral infection [46]. Also, Vázquez-Calvo and co-workers reported the probable use of VPA in understanding the crucial steps of viral maturation and for developing potent inhibitor for enveloped viruses [47].

## Conclusions

It is concluded that *L. cephalotes* extracts and oleanolic acid had significant anti-dengue activity on tested cell lines. Molecular docking conducted to validate the results against the dengue virus shows the maximum binding energy to dengue protein. The preliminary results obtained from in vitro and in silico studies are promising. Thus, oleanolic acid could be a source for drug design for the treatment of dengue as an antiviral agent.

## Supplementary Information

**Additional file 1.**

**Additional file 2.**

**Additional file 3.**

**Additional file 4.**

## Data Availability

All data generated or analysed during this study are included in this published article [and its supplementary information files].

## Abbreviations

SFE: Supercritical fluid extraction
UV-Vis: Ultraviolet-visible spectroscopy
FT-IR: Fourier transform infra-Red spectroscopy
HPTLC: High-performance thin-layer chromatography
^1^HNMR/^13^CNMR: Proton/carbon nuclear magnetic resonance
MNTD: Maximum non-toxic dose
DENV: Dengue virus
DMSO: Dimethyl sulfoxide
MEM: Minimum essential medium
MTT: 3–4, 5-dimethylthiazol-2, yl-2,5- diphenyltetrazolium bromide
PBS: Phosphate buffer saline
FCS: Fetal calf serum
