# Supplementary figures and images for: Anti-dengue activity of super critical extract and isolated oleanolic acid of Leucas cephalotes using in vitro and in silico approach
Source: BMC Complement Med Ther. 2021 Sep 8;21:227. doi: 10.1186/s12906-021-03402-2 (PMC8425015; doi:10.1186/s12906-021-03402-2)

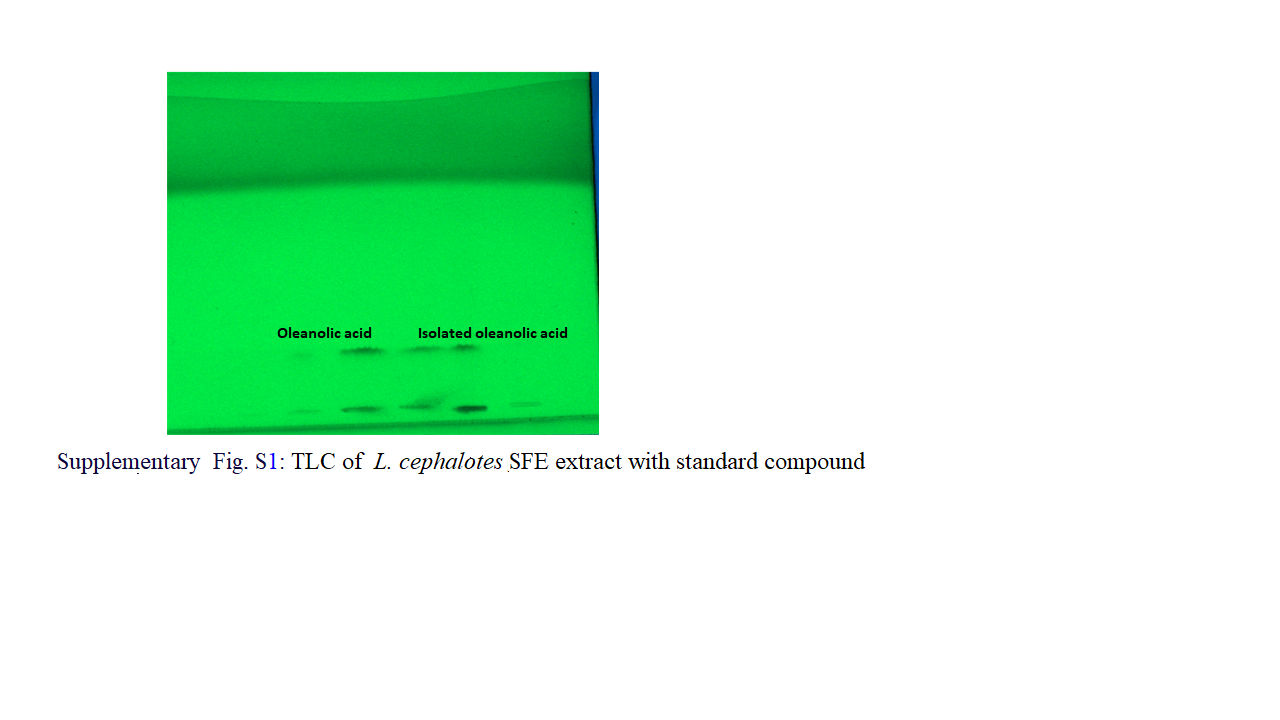

Supplement: Supplementary file 1 — Additional file 1. [file 12906_2021_3402_MOESM1_ESM.tif]

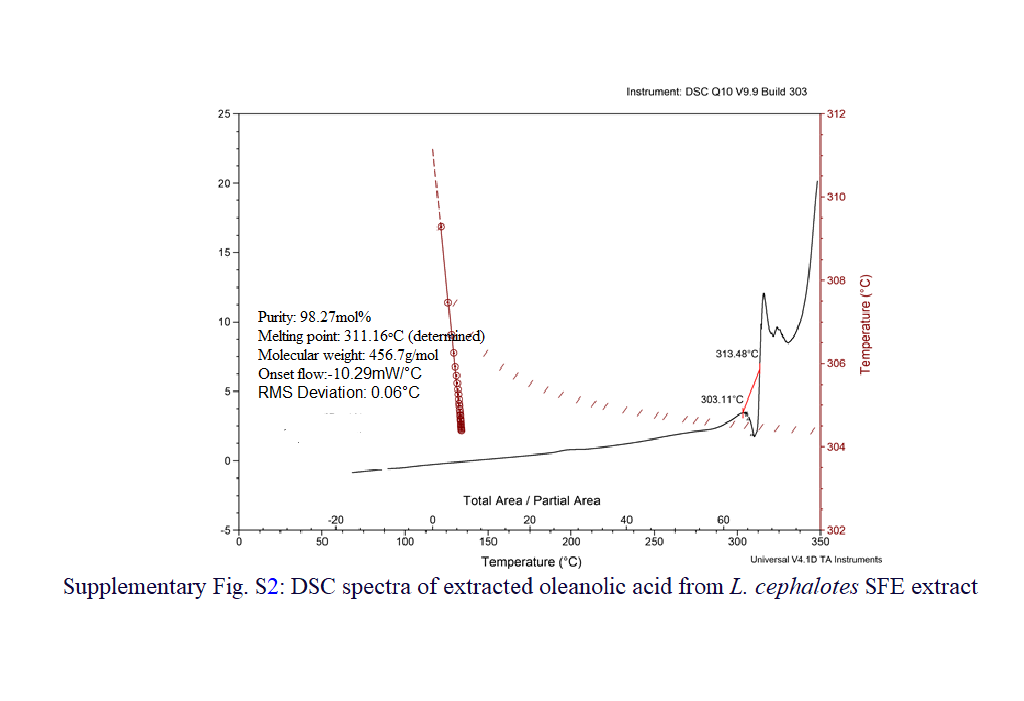

Supplement: Supplementary file 2 — Additional file 2. [file 12906_2021_3402_MOESM2_ESM.tif]

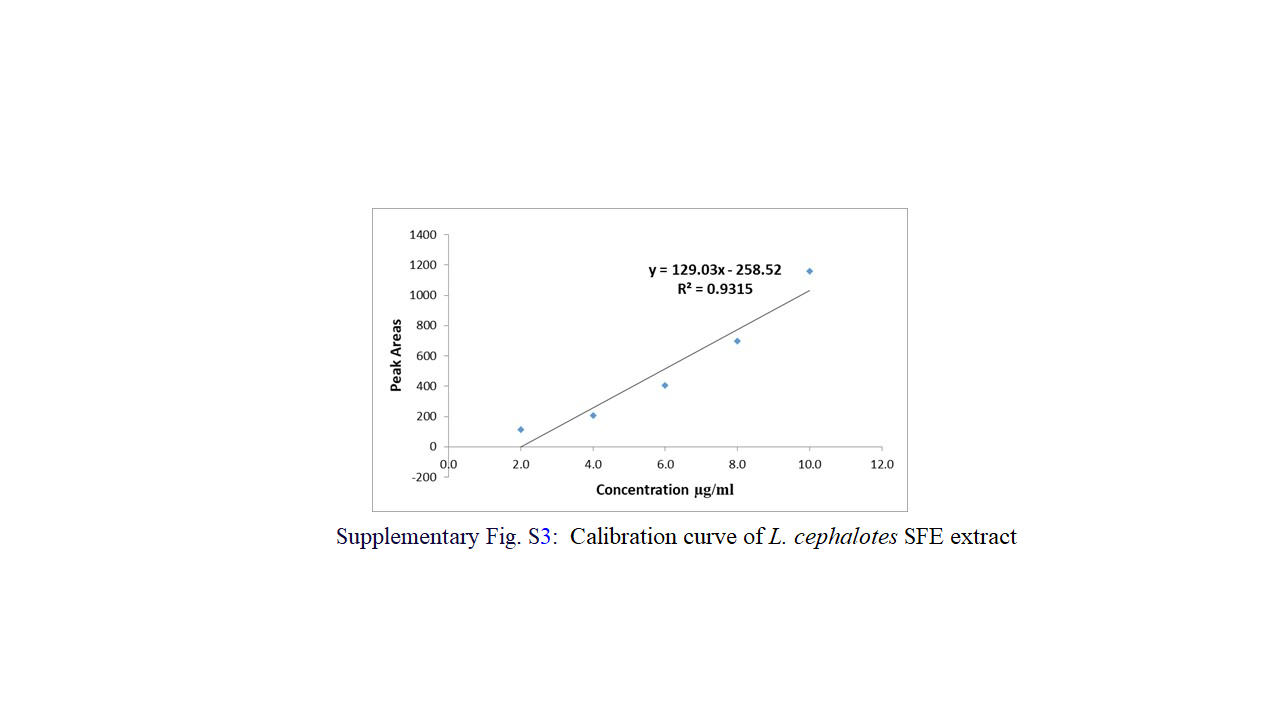

Supplement: Supplementary file 3 — Additional file 3. [file 12906_2021_3402_MOESM3_ESM.tif]

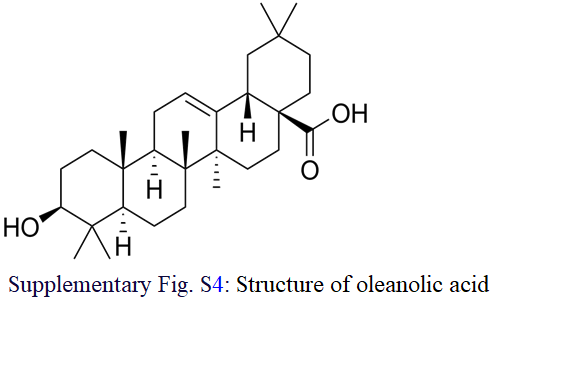

Supplement: Supplementary file 4 — Additional file 4. [file 12906_2021_3402_MOESM4_ESM.tif]
